# The angiotensin-converting enzyme I/D polymorphism does not impact training-induced adaptations in exercise capacity in patients with stable coronary artery disease

**DOI:** 10.1038/s41598-023-45542-0

**Published:** 2023-10-25

**Authors:** Tórur Sjúrðarson, Jacobina Kristiansen, Nikolai B. Nordsborg, Noomi O. Gregersen, Leivur N. Lydersen, Erik L. Grove, Steen D. Kristensen, Anne-Mette Hvas, Magni Mohr

**Affiliations:** 1 Center of Health Science, Faculty of Health, University of the Faroe Islands, Tórshavn, Faroe Islands; 2 Department of Nutrition, Exercise and Sports (NEXS), University of Copenhagen, Copenhagen, Denmark; 3 Department of Medicine, National Hospital of the Faroe Islands, Tórshavn, Faroe Islands; 4 Department of Clinical Biochemistry, Aarhus University Hospital, Aarhus, Denmark; 5 Department of Cardiology, Aarhus University Hospital, Aarhus, Denmark; 6 Faculty of Health, Aarhus University, Aarhus, Denmark; 7 FarGen, the Faroese Health Authority, Tórshavn, Faroe Islands; 8 Department of Sports Science and Clinical Biomechanics, SDU Sport and Health Sciences Cluster (SHSC), Faculty of Health Sciences, University of Southern Denmark, 5250 Odense, Denmark

**Keywords:** Physiology, Cardiology, Diseases

## Abstract

Systematic exercise training effectively improves exercise capacity in patients with coronary artery disease (CAD), but the magnitude of improvements is highly heterogeneous. We investigated whether this heterogeneity in exercise capacity gains is influenced by the insertion/deletion (I/D) polymorphism of the angiotensin-converting enzyme (ACE) gene. Patients with CAD (n = 169) were randomly assigned to 12 weeks of exercise training or standard care, and 142 patients completed the study. The ACE polymorphism was determined for 128 patients (82% males, 67 ± 9 years). Peak oxygen uptake was measured before and after the 12-week intervention. The ACE I/D polymorphism frequency was n = 48 for D/D homozygotes, n = 61 for I/D heterozygotes and n = 19 for I/I homozygotes. Baseline peak oxygen uptake was 23.3 ± 5.0 ml/kg/min in D/D homozygotes, 22.1 ± 5.3 ml/kg/min in I/D heterozygotes and 23.1 ± 6.0 ml/kg/min in I/I homozygotes, with no statistical differences between genotype groups (*P* = 0.50). The ACE I/D polymorphism frequency in the exercise group was n = 26 for D/D, n = 21 for I/D and n = 12 for I/I. After exercise training, peak oxygen uptake was increased (*P* < 0.001) in D/D homozygotes by 2.6 ± 1.7 ml/kg/min, in I/D heterozygotes by 2.7 ± 1.9 ml/kg/min, and in I/I homozygotes by 2.1 ± 1.3 ml/kg/min. However, the improvements were similar between genotype groups (time × genotype, *P* = 0.55). In conclusion, the ACE I/D polymorphism does not affect baseline exercise capacity or exercise capacity gains in response to 12 weeks of high-intensity exercise training in patients with stable CAD.

Clinical trial registration: www.clinicaltrials.gov (NCT04268992).

## Introduction

Coronary artery disease (CAD) is a leading cause of death worldwide^1^, and exercise training is an effective adjunct to the pharmacological treatment and rehabilitation of patients with CAD^2^. Exercise capacity, determined as whole-body peak oxygen uptake (VO_2peak_), is a robust and independent predictor of cardiac and all-cause mortality in patients with CAD^3^, and is readily upregulated with systematic exercise training.

Importantly, there is substantial inter-individual variability in the response to systematic exercise training, with training-induced improvements in VO_2peak_ ranging from no gains to improvements of more than 600 ml/min in patients with CAD^4^. Moreover, a recent study in 53 heart failure patients demonstrated a substantial prevalence of non-responders in VO_2peak_ gains and its central and peripheral determinants following 20 sessions (4–6 weeks) of exercise training^5^. Specifically, the prevalence of non-responders (defined as a relative increase < 10%) was 45% for VO_2peak_, 32% for maximal cardiac output and 44% for maximal oxygen extraction capacity, but only four patients were classified as non-responders on all three variables^5^. Thus, it is of great importance to investigate potential factors contributing to this inter-individual variation in exercise capacity trainability.

A potentially decisive determinant of exercise capacity adaptability is the insertion (I) and deletion (D) polymorphism in the human angiotensin-converting enzyme (ACE) gene, which is located on chromosome 17 at heading 23 (17q23)^6–8^. Circulating levels of ACE are largely determined by the I/D polymorphism, with the I allele associated with low plasma ACE levels and the D allele with high plasma ACE levels^6^.

Importantly, an effect of the ACE I/D polymorphism on VO_2peak_ trainability has been observed in patients with CAD. Defoor et al.^9^ reported that patients with the I/I genotype demonstrated significantly greater VO_2peak_ gains compared to carriers of the D allele in response to 3 months of exercise training in 933 Caucasian patients diagnosed with CAD. Patients with CAD are commonly recommended treatment with ACE inhibitors^10^, which have been proven to reduce serum ACE levels^11^ and thereby mimic the ACE phenotype categorised by the ACE I/I homozygotes. The impact of ACE inhibitor treatment on circulating ACE levels may be ACE genotype-specific, with one study demonstrating a significantly larger decrease in plasma ACE level in patients with the D/D genotype compared to patients with the I/I genotype^12^. Interestingly, Defoor et al.^9^ also performed a sub-analysis excluding patients treated with ACE inhibitors (n = 688), which resulted in a more pronounced difference in VO_2peak_ improvement between the I/I homozygotes and the D/D homozygotes. Moreover, Abraham et al.^13^ observed a significantly higher baseline VO_2peak_ in 57 patients with congestive stable heart failure having the I/I genotype compared to carriers of the D allele, and the ACE I allele has been extensively linked to enhanced endurance performance in healthy individuals^8,14^. To date, the underlying physiological mechanisms remain largely unresolved, but accumulating evidence suggests that the potential association between the ACE genotype and performance-related phenotypes is related to ACE genotype-dependent modulations of muscular efficiency^15–20^, plausibly through ACE activity-dependent regulation of local nitric oxide bioavailability, which has been demonstrated to influence mitochondrial respiration and, consequently, metabolic efficiency^21,22^.

Given the potential link between the ACE genotype and exercise capacity trainability in patients with CAD, the present study was undertaken to determine whether the ACE I/D genotype affects the outcome of systematic supervised exercise training on exercise capacity in patients with CAD.

## Methods

### Patients and study design

A total of 169 patients older than 18 years with angiographically confirmed CAD were recruited for the study, and parts of the obtained data have been reported elsewhere^4,23^. Patients were randomly assigned 1:1 to supervised high-intensity interval training (HIIT) or standard care. Inclusion and exclusion criteria as well as the randomisation procedure have been described in detail previously^4^. All patients provided written informed consent after receiving written and oral information about the study protocol and associated risks. A total of 142 patients completed the study, and the ACE genotype was determined in 128 patients (Table 1). The study was approved by the ethics committee and data inspectorate of the Faroe Islands and conducted in accordance with the Declaration of Helsinki. The study protocol was registered at ClinicalTrials.gov (Identifier: NCT04268992). Exercise capacity measurements were obtained before and after 12 weeks of supervised exercise training at the National Hospital of the Faroe Islands.

**Table 1:** Patient characteristics.

Outcome	All (n = 128)	ACE D/D (n = 48)	ACE I/D (n = 61)	ACE I/I (n = 19)	*P* value DD versus ID versus II
Age (years)	67 ± 9	66 ± 9	67 ± 10	69 ± 11	0.46
Height (cm)	174 ± 8	174 ± 7	175 ± 8	174 ± 8	0.93
Weight (kg)	90.5 ± 17.3	89 ± 17	93 ± 18	87 ± 17	0.42
BMI	29.7 ± 4.9	29.2 ± 5.0	30.4 ± 4.8	28.6 ± 5.2	0.29
Gender (male/female)	105/23 (82%/18%)	41/7 (85%/15%)	49/12 (80%/20%)	15/4 (79%/19%)	0.77
Medication					
ACE inhibitor	51 (40%)	23 (48%)	21 (34%)	7 (37%)	0.43

Anthropometrics are presented as means ± standard deviation, and dichotomous data are expressed as numbers (percentages) in patients with the ACE D/D, ACE I/D or ACE I/I genotype. BMI: body mass index. The *P* value of the one-way ANOVA is presented.

### Exercise training

The prescribed exercise training programme has been described in detail previously^4^. Briefly, the supervised exercise training was performed as HIIT training three times a week for 12 weeks on an indoor rowing ergometer (Concept 2 model D w. PM5, Vermont, United States). Each exercise session consisted of a 6-min warm-up followed by high-intensity interval training, which averaged an active training time of 12 min per session. Power output was monitored in weeks three, six and nine, and the average relative power output was determined as the average power output normalized to the average power during a 5 min all‐out rowing‐ergometer effort performed on week 5. Compliance with the exercise sessions was 97%, with an overall range of 86–100%.

### ACE genotyping

Determination of the patients’ ACE genotype was performed as previously described^24,25^. Briefly, genomic DNA was extracted from whole-blood samples by means of the chemagic Prepito-D platform (PerkinElmer, Waltham, MA, USA). The SYBR Green I PCR Master Mix reagents (Applied Biosystems, MA, USA) were used for real-time amplification of the I/D polymorphism in intron 16 of the ACE gene. Primer design, amplification and detection of the ACE I/D polymorphism were performed as previously described^24,26,27^, using SYBR Green I PCR Master Mix reagents (Applied Biosystems, MA, USA) on the StepOnePlus™ Real-Time PCR System (Applied Biosystems, MA, USA) and followed by a melting curve analysis according to the manufacturer’s protocol (Applied Biosystems, MA, USA).

### Exercise capacity measurements

Patients reported to the exercise laboratory in a fasted state (> 1.5 h) and were explicitly told to refrain from tobacco, caffeine and alcohol on testing day and to avoid strenuous exercise for at least 24 h prior to exercise testing.

Peak oxygen uptake (VO_2peak_) and peak workload were determined on a cycle ergometer (Excalibur Sport, Lode, Groningen, Netherlands) as previously described^4^. In brief, the exercise protocol included a 6-min standardized warm-up comprising 3 min at 30 W for females and 50 W for males, followed by 3 min at 50 W for females and 70 W for males. Subsequently, the workload was increased incrementally by 15 W/min for females and 20 W/min for males until exhaustion. Cycling peak workload was registered and oxygen uptake was measured continuously throughout the exercise test (model Cosmed, Quark b2, Milano, Italy). In addition, heart rate was monitored continuously throughout the exercise protocol (HRM-Dual, Garmin, Olathe, Kansas, USA). Peak oxygen uptake was defined as the highest 30s average recorded. Possible ACE genotype-dependent changes in muscular efficiency with training were evaluated using steady-state VO_2_ at fixed submaximal workloads as previously done by Woods et al.^16^. Steady-state VO_2_ was determined as the average oxygen uptake during the final 30 s of each warm-up interval, and steady-state heart rate was determined as the average heart rate during the final 30 s of the warm-up bout.

The exercise protocol was conducted on a cycle ergometer to ensure that potential training-induced adaptations in exercise capacity and muscular efficiency were induced by physiological adaptations to the applied training intervention rather than familiarization with the rowing ergometer and/or improved rowing technique.

### Statistics

Anthropometric and baseline exercise capacity measurements are presented as means with standard deviation and compared using a one-way ANOVA. Proportions are expressed in percentages and compared by Pearson’s chi-squared test if model assumptions were met and otherwise by Fisher’s exact test.

Changes in exercise capacity and steady-state VO_2_ in response to the intervention are presented as means with standard deviation and analysed by means of a repeated-measures mixed model using the SPSS mixed procedure (SPSS statistics v. 28.0.0, IBM)^28^, which included time (pre vs post), genotype (D/D vs. I/D vs. ID) and a time × genotype interaction as explanatory factors. Potential difference in response to exercise training between genotype groups was evaluated by the interaction effect. Significant main effects for ‘time’, ‘genotype’ or ‘time × genotype’ interactions were further assessed by Sidak-adjusted pairwise comparisons. The independence of residuals was assumed in the model. Normal distribution of residuals and equal variance of residuals were visually inspected. No clear violations of the model assumptions existed.

Finally, potential genotype-specific differences in average power output and relative power output during the exercise training sessions were assessed by a one-way ANOVA (D/D vs I/D vs I/I). Power output is presented as means with standard deviations. The level of statistical significance was set at *P* < 0.05.

## Results

The ACE I/D genotype distribution was n = 48 for D/D, n = 61 for I/D and n = 19 for I/I. As illustrated in Table 1, patients with the D/D, I/D and I/I genotype were comparable in all obtained anthropometric measures as well as in gender distribution and proportion of ACE inhibitor treatment.

### ACE genotype and pre-training exercise capacity

All obtained measures of exercise capacity were similar between genotype groups at baseline (Table 2). In addition, a sub-analysis was performed without ACE inhibitor users (n = 76), but no significant between-groups differences were observed (Table 2).

**Table 2:** Baseline exercise capacity.

Outcome	All (n = 128)	ACE D/D (n = 48)	ACE I/D (n = 61)	ACE I/I (n = 19)	*P* value DD versus ID versus II
VO_2peak_ (mL/kg/min)	22.7 ± 5.3	23.3 ± 5.0	22.1 ± 5.3	23.1 ± 6.0	0.50
VO_2peak_ (mL/min)	2036 ± 553	2040 ± 442	2048 ± 619	1990 ± 605	0.93
Cycling peak power (W)	160 ± 54	165 ± 45	158 ± 59	152 ± 61	0.63
Steady-state VO_2_ (mL/min)	1409 ± 215	1403 ± 205	1435 ± 221	1344 ± 215	0.27

Without ACE inhibition	All (n = 76)	ACE D/D (n = 25)	ACE I/D (n = 40)	ACE I/I (n = 11)	*P* value DD versus ID versus II
VO_2peak_ (mL/kg/min)	22.9 ± 5.5	23.5 ± 5.3	22.0 ± 5.4	25.2 ± 6.0	0.19
VO_2peak_ (mL/min)	2086 ± 588	2175 ± 453	2018 ± 664	2129 ± 586	0.56
Cycling peak power (W)	164 ± 58	176 ± 47	155 ± 63	170 ± 58	0.31
Steady-state VO_2_ (mL/min)	1410 ± 202	1440 ± 234	1402 ± 189	1371 ± 180	0.61

Values are presented as means ± standard deviations for patients with the ACE D/D, I/D or I/I genotype. The *P* value of the one-way ANOVA is presented.

### Exercise training

For the patients allocated to exercise training, the ACE genotype distribution was n = 26, n = 21 and n = 12 for D/D, I/D and I/I, respectively. Patients with the ACE D/D, ACE I/D or ACE I/I genotype had similar gender and ACE inhibitor treatment distribution and were comparable in age, height, weight and BMI (Table 3).

**Table 3:** Patients allocated to exercise training.

Outcome	All (n = 59)	ACE D/D (n = 26)	ACE I/D (n = 21)	ACE I/I (n = 12)	*P* value DD versus ID versus II
Age (years)	67 ± 10	65 ± 9	67 ± 9	70 ± 10	0.31
Height (cm)	174 ± 7	174 ± 7	175 ± 8	173 ± 7	0.78
Weight (kg)	89 ± 17	88 ± 15	93 ± 18	85 ± 20	0.40
BMI	29.4 ± 4.9	29.1 ± 4.5	30.4 ± 4.5	28.4 ± 6.3	0.47
Gender (male/female)	49/10 (83%/17%)	22/4 (85%/15%)	18/3 (86%/14%)	9/3 (75%/25%)	0.74
Medication					
ACE inhibitor	29 (49%)	14 (54%)	9 (43%)	6 (50%)	0.75

Anthropometrics are presented as means ± standard deviation, and dichotomous data are expressed as numbers (percentages) in patients with the ACE D/D, ACE I/D or ACE II genotype. BMI: body mass index. The *P* value of the one-way ANOVA is presented.

### Exercise power output and relative intensity

The average power output during the exercise intervals was similar between genotype groups (D/D: 139 ± 39 W vs. I/D: 139 ± 54W vs. I/I: 120 ± 42 *P* = 0.45). Accordingly, no between-group difference was observed for the average relative power output during the exercise intervals (D/D: 117 ± 9% vs. I/D 116 ± 9% vs. I/I 122 ± 18, *P* = 0.32), which was determined as the average power output during weeks three, six and nine normalized to the average power output during a maximal 5-min rowing effort performed in week 5 of the intervention.

### ACE genotype and exercise capacity improvements

**Figure 1:**
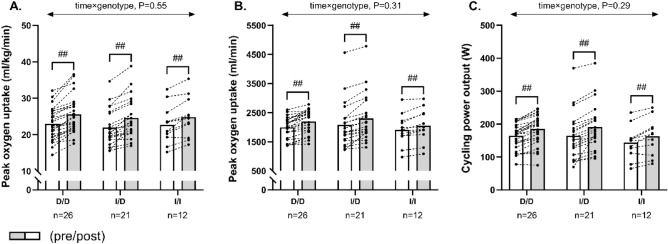
The figure shows mean values for peak oxygen uptake adjusted for body weight (**A**), absolute peak oxygen uptake (**B**) and cycling peak power (**C**) in histograms with individual participants represented as lines among D/D homozygotes, I/D heterozygotes and I/I homozygotes, measured pre (white bars) and post (grey bars) 12 weeks of exercise training. The *P* interaction value of the linear mixed-model with time, genotype, and time × genotype as fixed factors is presented. ## Denotes within-group difference from baseline at *P* < 0.001.

**Figure 2:**
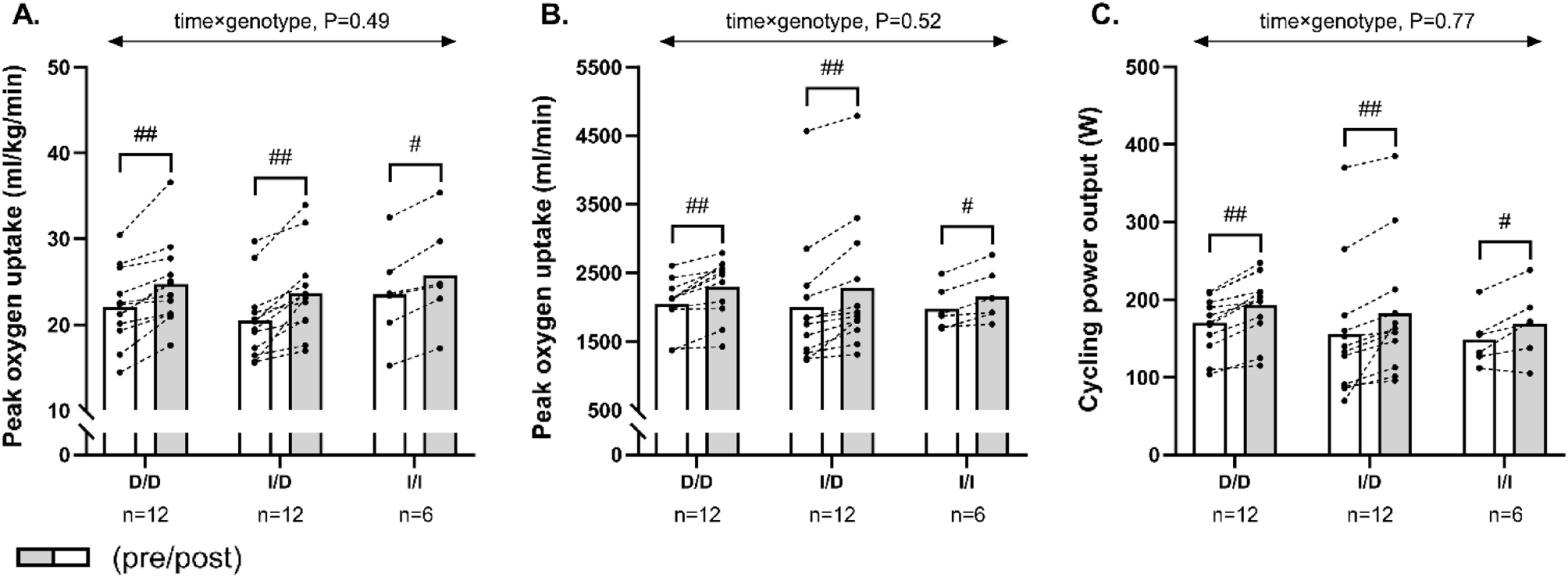
The figure shows mean values for peak oxygen uptake adjusted for body weight (**A**), absolute peak oxygen uptake (**B**) and cycling peak power (**C**) in histograms with individual participants represented as lines among D/D homozygotes, I/D heterozygotes and I/I homozygotes not treated with ACE inhibitors, measured pre (white bars) and post (grey bars) 12 weeks of exercise training. The *P* interaction value of the linear mixed-model with time, genotype, and time × genotype as fixed factors is presented. #, ## Denotes within-group difference from baseline at *P* < 0.05 and *P* < 0.001, respectively.

Twelve weeks of exercise training effectively increased (*P* < 0.001) VO_2peak_ adjusted for body weight, absolute VO_2peak_ and cycling peak power in all ACE genotype groups, with magnitudes of 2.6 ± 1.7 ml/kg/min, 205 ± 140 ml/min and 21 ± 12 W in D/D homozygotes, of 2.7 ± 1.9 ml/kg/min, 222 ± 160 ml/min and 27 ± 19 W in I/D heterozygotes, and 2.1 ± 1.3 ml/kg/min, 144 ± 105 ml/min and 19 ± 14 W in I/I homozygotes(Fig. 1). The improvements were similar between genotype groups for all obtained measures of exercise capacity (time × genotype, *P* ≥ 0.29; Fig. 1). When excluding patients treated with ACE inhibitors in a sub-analysis, VO_2peak_ adjusted for body weight, absolute VO_2peak_ and cycling peak power increased (*P* < 0.01) by 2.6 ± 1.7 ml/kg/min, 236 ± 155 ml/min and 24 ± 11 W amongst D/D homozygotes, by 3.2 ± 2.1 ml/kg/min, 266 ± 189 ml/min and 27 ± 25W in I/D heterozygotes, and by 2.3 ± 1.0 ml/kg/min, 172 ± 101 ml/min and 20 ± 17 in I/I homozygotes, but no significant between-group effect could be demonstrated (time × genotype, P ≥ 0.49; Fig. 2).

### ACE genotype and steady-state VO_2_ and heart rate

**Figure 3:**
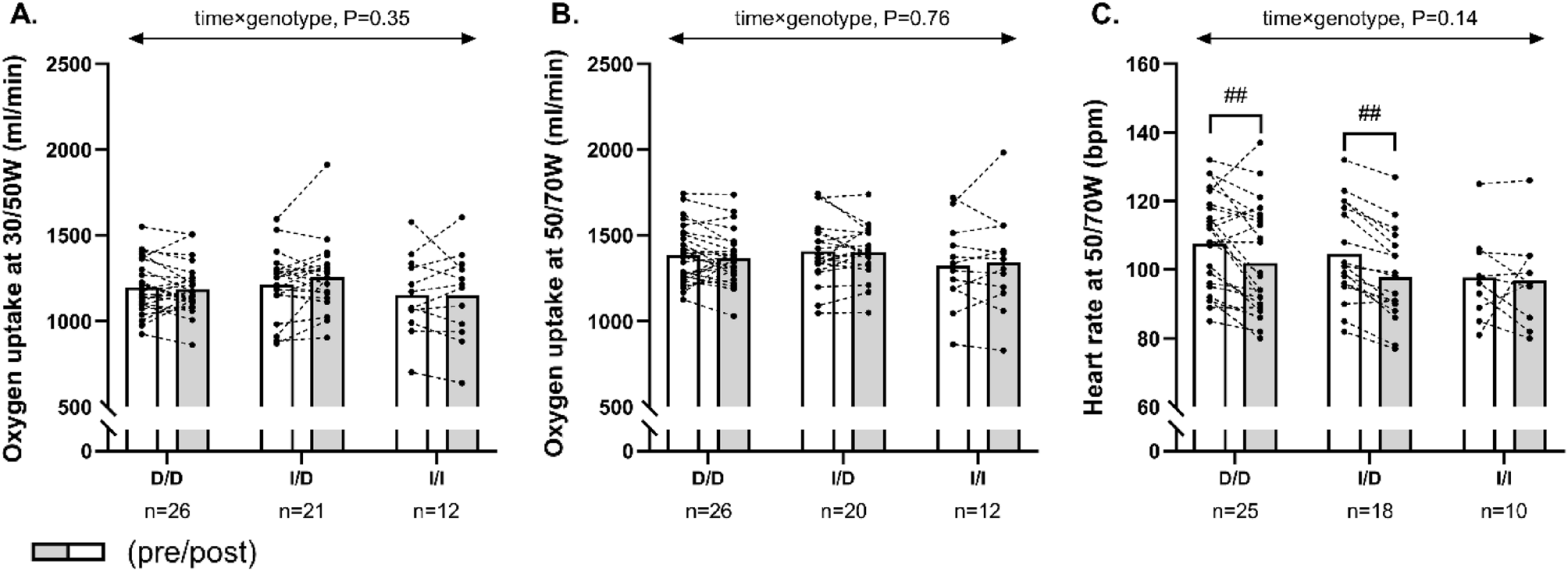
The figure shows mean values for oxygen uptake at 30/50W (females/males) (**A**), oxygen uptake at 50/70W (females/males) (**B**) and heart rate at 50/70W (females/males) (**C**) in histograms with individual participants represented as lines among D/D homozygotes, I/D heterozygotes and I/I homozygotes, measured pre (white bars) and post (grey bars) 12 weeks of exercise training. The *P* interaction value of the linear mixed-model with time, genotype, and time × genotype as fixed factors is presented. ## Denotes within-group difference from baseline at *P* < 0.001.

**Figure 4:**
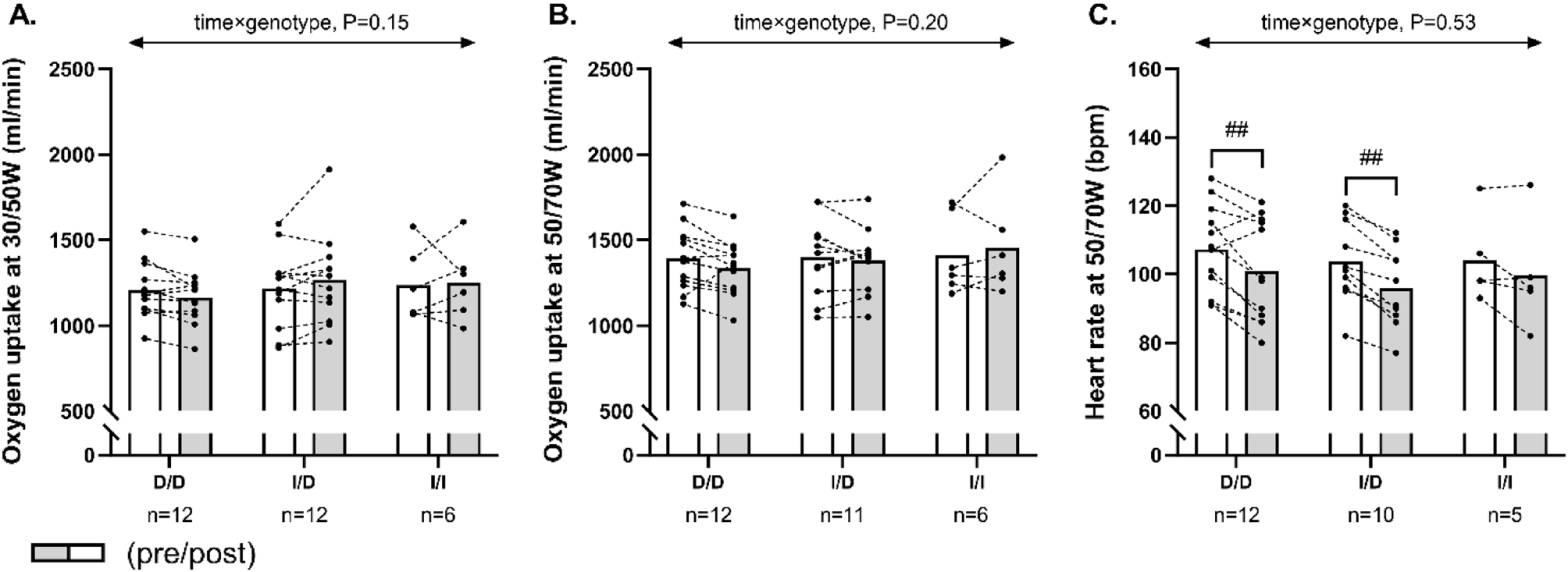
The figure shows mean values for oxygen uptake at 30/50W (females/males) (**A**), oxygen uptake at 50/70W (females/males) (**B**) and heart rate at 50/70W (females/males) (**C**) in histograms with individual participants represented as lines among D/D homozygotes, I/D heterozygotes and I/I homozygotes not treated with ACE inhibitors, measured pre (white bars) and post (grey bars) 12 weeks of exercise training. The P interaction value of the linear mixed-model with time, genotype, and time × genotype as fixed factors is presented. ## Denotes within-group difference from baseline at *P* < 0.001.

No significant within-group or between-group effect existed for changes in steady-state VO_2_ at fixed submaximal workloads (Fig. 3A,B). Furthermore, no between-group effect was observed for changes in steady-state HR (Fig. 3C), but the post-hoc analysis demonstrated a significant training-induced reduction (*P* < 0.001) in steady-state heart rate of − 6 ± 8 bpm in D/D homozygotes and of − 7 ± 4 bpm in I/D heterozygotes, whereas no changes were observed in I/I homozygotes (− 0.8 ± 10 bpm, *P* = 0.73; Fig. 3C). Excluding ACE inhibitor users from the analysis did not significantly modify any of the obtained measures (Fig. 4).

### Carriers of the I allele versus D/D homozygotes

Due to the low frequency of ACE I/I homozygotes and to ensure a more balanced genotype distribution in the statistical analysis, a sub-analysis, in which carriers of the I allele were combined into a single group (I/ +), was conducted. Patients with the ACE D/D or I/ + genotype had similar gender and ACE inhibitor treatment distribution and were comparable in age, height, weight and BMI (Table 4).

**Table 4:** Patients allocated to exercise training.

Outcome	All (n = 59)	ACE D/D (n = 26)	ACE I/ + (n = 33)	*P* value DD versus I +
Age (years)	67 ± 10	65 ± 10	68 ± 9	0.17
Height (cm)	174 ± 7	174 ± 7	174 ± 8	0.92
Weight (kg)	89 ± 17	88 ± 16	90 ± 19	0.66
BMI	29.4 ± 4.9	29.1 ± 4.6	29.6 ± 5.2	0.66
Gender (male/female)	49/10 (83%/17%)	22/4 (84%/16%)	27/6 (82%/18%)	1.00
Medication				
ACE inhibitor	29 (49%)	14 (54%)	15 (45%)	0.70

Anthropometrics are presented as means ± standard deviation, and dichotomous data are expressed as numbers (percentages) in patients with the ACE D/D or ACE I/ + genotype. BMI: body mass index. The *P* value of the independent samples t-test is presented.

Combining carriers of the I allele into one group did not significantly alter the results for any of the obtained markers of exercise capacity, with between-group *P*-values of *P* = 0.74 for changes in VO_2max_ adjusted for body weight, *P* = 0.77 for changes in absolute VO_2max_ and *P* = 0.42 for changes in cycling peak power. Similarly, no statistical between-group effect was observed for changes in steady-state oxygen uptake measurements and changes in steady-state heart rate, with between-group *P*-values of *P* = 0.34 for changes in oxygen uptake at 30/50W, *P* = 0.68 for changes in oxygen uptake at 50/70W and *P* = 0.65 for changes in heart rate at 50/70W. Excluding patients treated with ACE inhibitors from the analysis did not statistically alter any of the obtained measures.

## Discussion

We investigated the significance of the ACE I/D genotype for pre-training exercise capacity and whether the ACE I/D genotype impacts the outcome of 12 weeks of whole-body high-intensity interval training on exercise capacity in patients with stable CAD. The pre-training exercise capacity was independent of the ACE genotype. The applied low-volume high-intensity exercise protocol efficiently improved exercise capacity in the ACE D/D, ACE I/D and ACE I/I genotype groups, but the magnitude of improvements was similar between groups. Thus, our findings indicate no interaction between the ACE I/D genotype and pre-training exercise capacity and exercise capacity trainability in patients with CAD.

### ACE genotype and exercise capacity

Exercise capacity is a powerful predictor of the risk of all-cause mortality in patients with cardiovascular conditions^3^, and is inversely correlated with long-term mortality with apparently no upper limit of benefit in patient groups^29^. However, there is substantial inter-individual variability in exercise capacity trainability in both healthy individuals and patient populations^4,30,31^. Thus, the identification of potential predictors of exercise capacity adaptability is highly warranted.

In our study, we assessed the impact of the ACE I/D genotype on baseline exercise capacity and training-induced adaptations in exercise capacity, since previous research has reported that the I/I genotype is associated with greater baseline exercise capacity^13^ and exercise capacity trainability^9^ compared to the D/D genotype in patients diagnosed with cardiovascular disease. In contrast to the findings by Abraham et al.^13^, baseline exercise capacity was independent of the patients’ ACE genotype in the present study. Despite the low training volume of only ~ 18 min of effective training time per session, we observed marked training-induced improvements in exercise capacity in all ACE genotype groups. However, the findings by Defoor et al.^9^ could not be replicated in the present study as patients with the D/D, I/D and I/I genotype demonstrated similar improvements in exercise capacity in response to the prescribed exercise training intervention. The reasons for the discrepancy between the results of the present study and the study by Defoor et al.^9^ are unknown but could be related to the applied training intervention. Indeed, Defoor et al.^9^ utilized continuous aerobic training at a moderate intensity (79.9% of HR_max_) lasting 90 min per session^32^, whereas low volume HIIT was applied in the present study. Low-volume HIIT was applied in the present study because adhering to time-consuming exercise training may be challenging for elderly CAD patients, and meta-analysis data have confirmed the feasibility and superior cardiorespiratory benefits of HIIT compared to moderate-intensity continuous training in patients with CAD^33–36^. Moreover, the patients in the study by Defoor et al.^9^ were more than 10 years younger than the patients in the present study (56 vs. 67 years), and some evidence indicates an age-related decline in VO_2max_ trainability^4^. Importantly, it should be noted that Defoor et al.^9^ measured their patients’ exercise capacity using two different gas analysing systems: an Oxycon Alphaw (Jaeger, Mijnhardt, Bunnik, the Netherlands) until 1994 and a 2900Zw (Sensormedics, Bilthoven, the Netherlands) from 1994 to 2001, constituting a major limitation and, consequently, the results by Defoor et al. should be interpreted with care. Notably, our results should also be interpreted with caution due to the significantly lower sample size (n_exercise_ = 59 patients) compared to Defoor et al.^9^ (n = 933). Indeed, a critical attribute of a research study is its inherent statistical power to detect a true effect or a true difference. In this context, previous estimates have shown that a sample size of ≥ 800 participants is commonly required in genetic/genomic association studies to gain sufficient power to detect associations that account for ~ 1% of the variability of a particular trait^37^. Given that the ACE genotype is only one of a myriad of potential genetic and environmental determinants of the inter-individual variation in the trainability of the complex exercise-related trait, *VO*_*2max*_, it is reasonable to assume that the present study has an inadequate level of statistical power to definitively reject an effect of the ACE genotype on exercise capacity trainability. However, the present findings do not indicate a clinically important role for the ACE genotype as a modulator of exercise capacity trainability in this patient group. Furthermore, the recruitment of a heterogeneous patient population with comorbidities and simultaneous prescription of a high number of medical treatments^4^, which might themselves interact with the ability to adapt to exercise training might also have masked a potential effect of the ACE genotype. For instance, more than 90% of the patients included in the present study were treated with statins^4^, which may have adverse effects on skeletal muscle mitochondrial content and oxidative capacity^38–41^ and have been shown to abolish exercise training-induced upregulation in muscle mitochondrial biogenesis as well as exercise capacity^38^. Moreover, 40% of the patients in our study were prescribed ACE inhibitors, and 49% of the patients allocated to exercise training were ACE inhibitor users (Table 4). Interestingly, Defoor et al.^9^ demonstrated that excluding patients treated with ACE inhibitors from the statistical analysis increased the magnitude of differences in training-induced exercise capacity gains between the ACE I/I homozygotes and the D/D homozygotes from 2.1 to 3.0%. As stated in the introduction, the ACE I allele is associated with low serum ACE levels, whereas the ACE D allele is associated with high serum ACE levels^6,12^, and pharmacological ACE inhibition effectively lowers serum ACE levels^11^. Thus, it seems reasonable to speculate that pharmacological ACE inhibition may mitigate the differences in exercise capacity trainability between the ACE genotype groups by mimicking the ACE phenotype categorized by the I allele in ACE D/D homozygotes. In support of this rationale, Todd et al.^12^ demonstrated that a single dose (10 mg) of the ACE inhibitor enalapril induced a significantly greater reduction in serum ACE levels in D/D compared to I/I homozygotes^12^. If the association between the I allele and endurance performance is related to lower basal levels of circulating ACE, then a synergistic effect of ACE inhibitor treatment on training-induced improvements in exercise capacity seems plausible, especially for D/D homozygotes. To assess whether the ACE I/D genotype affects the interaction between ACE inhibitor treatment and exercise capacity trainability, we conducted a sub-analysis on the patients treated with ACE inhibitors, which constituted time, genotype, ACE_inhibitor_treatment and a time × genotype × ACE_inhibitor_treatment interaction as fixed factors. However, the time × genotype × ACE_inhibitor_treatment interaction did not reach statistical significance, indicating that exercise capacity trainability in patients treated with ACE inhibitors does not appear to be affected by their ACE genotype (data not shown). In general, studies assessing the impact of ACE inhibitor treatment on markers of exercise capacity in patients have yielded conflicting results. Indeed, a positive impact of ACE inhibitor treatment alone on exercise capacity^42,43^ as well as a synergistic effect of ACE inhibitor treatment on exercise responsiveness has been reported^44^, whilst other studies have observed no effect or adverse effects of pharmacological ACE inhibition on exercise capacity^24,45–47^. However, these studies have not accounted for the patients’ ACE I/D genotype.

Finally, we also performed a sub-analysis to assess the effect of the ACE genotype on baseline exercise capacity as well as exercise capacity trainability for the patients not treated with ACE inhibitors. Excluding ACE inhibitor users from the analysis did not significantly modify any of the obtained measures of exercise capacity, and hence no significant between-group effect was observed for pre-training exercise capacity or the magnitude of exercise capacity improvements. Our findings should, however, be interpreted with care because the low sample size in conjunction with the large variations in exercise capacity measurements augments the risk of undetected effects.

### ACE genotype and muscular efficiency

Muscular efficiency is critical for endurance performance^48^, and it seems plausible that the potential link between the ACE genotype and endurance-based phenotypes is related to ACE genotype-dependent alterations in muscular efficiency^16,17^. For instance, a training study in British military recruits reported that only participants with the ACE I/I genotype improved skeletal muscle efficiency following 11 weeks of exercise training, resulting in a significantly greater reduction in submaximal VO_2_ in I/I compared to D/D homozygotes^16,17^. Interestingly, the differences in muscular efficiency were not accompanied by ACE genotype-dependent differences in VO_2max_ response. As for potential mechanisms, inter-individual differences in serum ACE activity may affect the bioavailability of nitric oxide, which has been demonstrated to influence mitochondrial respiration and thus metabolic efficiency^21,22^. Bradykinin, whose levels are inversely related to serum ACE activity^49^, regulates the bioavailability of nitric oxide^50,51^, and hence, ACE inhibitor treatment has been shown to increase bradykinin levels and promote nitric oxide accumulation^52,53^. Notably, nitric oxide donors reportedly reduce mitochondrial VO_2_ in both myocytes^54,55^ and cardiomyocytes^56^, whereas administration of nitric oxide synthesis inhibitors reportedly increases whole-body VO_2_^57^. Furthermore, administration of ACE inhibitors has been shown to induce a significant ~ 25% reduction in cardiac VO_2_ in dogs^58^. The apparent effect of nitric oxide on mitochondrial respiration may be related to its role in regulating the capacity of cytochrome c oxidase to utilize oxygen^21,22^. Given this, it may be speculated that the innate disposition towards low plasma ACE activity associated with the ACE I/I genotype^6^ augments local nitric oxide bioavailability, which in turn improves the efficiency of the mitochondrial respiration in myocytes and cardiomyocytes, which reportedly contain complete kallikrein-kinin systems^59,60^. Furthermore, carriers of the I allele reportedly have a higher proportion of type I fibers, which are more efficient in slow contraction^61^, and chronic treatment with ACE inhibitors induces a shift toward the fatigue-resistant MHC 1 isoform in heart failure patients^62^. Similar findings have been reported in animal studies^63–65^. The patients’ muscle fiber composition was not determined in the present study, but we assessed potential ACE genotype-dependent differences in muscle efficiency by measuring oxygen uptake at two fixed submaximal intensities pre-and post-intervention. Previous studies have substantiated that low-volume HIIT efficiently improves submaximal energy expenditure^66^. However, in contrast to the findings by Woods et al.^16^ and Williams et al.^17^ in healthy military recruits, we observed no between-group differences for changes in steady-state VO_2_ in response to the intervention. The reason for conflicting findings may be related to the heterogeneity of the recruited cohort. Indeed, an effect of the ACE genotype on exercise trainability has consistently been observed in homogenous military recruits^15–17,67–69^, where the variability in non-genetic factors including gender, training status, age, medical treatment, diseases, sleeping duration and eating behaviours that may themselves interact with the training response is low, and therefore the small effect of a single genetic variant is more likely to be detected.

## Strengths and limitations

It is a strength that we recruited a well-described clinical population in which improvement in exercise capacity is highly important. The adherence to the prescribed supervised exercise sessions was high and the drop-out was low, which is a strength. The sample size is relatively high in terms of a training study, but low in terms of differentiating between different genotype sub-groups, and hence our findings should be interpreted with caution due to potential statistical type II errors in the comparisons between genotype groups. Furthermore, the unequal genotype group sample sizes may represent a limitation, as this can negatively affect statistical power and Type I error rates^70^. The issue of imbalanced genotype group sample sizes was partially accounted for in the assessment of ACE genotype-dependent differences in training-induced exercise capacity gains by combining the ACE I/D and ACE I/I genotype groups into one group, and by performing Sidak-adjusted pairwise comparisons, which is considered conservative and can be used for unequal sample sizes with equal variances^71^. Moreover, the dosages and types of ACE inhibitors are unknown and hence were not controlled for. Also, in the patients allocated to exercise training, the I/I homozygotes were numerically 5 years older than the ACE D/D homozygotes, which may be a potential confounder, although the difference was not significant. Finally, only 23 out of the 128 patients enrolled in the study were females, and only 10 out of the 59 patients allocated to exercise training were females. This skewed gender ratio clearly influences the generalizability of the present findings.

## Conclusion

Both baseline exercise capacity and exercise capacity improvements following 12 weeks of whole-body low-volume high-intensity exercise training seem to be independent of the ACE genotype in patients with CAD. Thus, the present findings do not support a clinically important role for the ACE genotype as a modulator of intrinsic or acquired exercise capacity in this patient group.

## Data Availability

The datasets generated and/or analyzed during the current study are available from the corresponding author on reasonable request.

## Abbreviations

ACE: Angiotensin-converting enzyme
CAD: Coronary artery disease
D: Deletion
HIIT: High-intensity interval training
I: Insertion
VO_2peak_: Peak oxygen uptake
